# Negative correlation between bone mineral density and TSH receptor antibodies in long-term euthyroid postmenopausal women with treated Graves’ disease

**DOI:** 10.1186/1756-6614-6-11

**Published:** 2013-09-11

**Authors:** Monica A Ercolano, Monica L Drnovsek, Maria C Silva Croome, Monica Moos, Ana M Fuentes, Fanny Viale, Ulla Feldt-Rasmussen, Alicia T Gauna

**Affiliations:** 1 Endocrinology Division, Hospital Ramos Mejía, Buenos Aires, Argentina; 2 Department of Medical Endocrinology, Rigshospitalet, Copenhagen University, Copenhagen, Denmark

## Abstract

**Background:**

Thyrotoxicosis is a cause of secondary osteoporosis. High concentrations of triiodotironine (T3) in Graves’ disease stimulate bone turnover, but it is unclear if euthyroidism will always normalize bone metabolism. Thyrotropin (TSH) is known to affect directly the bone metabolism through the TSH receptor and TSH receptor antibodies (TRAb) may have an important role in bone turn-over.

The aim of our study was to determine, in pre and postmenopausal euthyroidism patients with previous overt hyperthyroidism due to Graves’ disease the bone mineral density (BMD) as well as factors that could affect BMD in each group, including TRAb.

**Methods:**

Cross-sectional, non-interventional study. Fifty-seven patients with previous hyperthyroidism due to Graves’ disease (premenopausal: 30, postmenopausal: 27) that remained euthyroid for at least 6 months prior to study were included and compared with fifty- two matched respective controls. Thyrotoxine (T4), TSH, TRAb and BMD were measured.

**Results:**

Only euthyroid postmenopausal patients with a history of hyperthyroidism due to Graves’ disease showed lower whole body BMD than matched controls. The BMD expressed as Z-score was less in whole body and lumbar spine in postmenopausal in relation to premenopausal women with previous overt hyperthyroidism due to Graves’ disease.

In the postmenopausal patients, the Z-score of lumbar spine BMD correlated negatively with TRAb (r = −0,53, p < 0.008), positively with the time of evolution of the disease (r = +0.42, p < 0.032) and positively with the time of euthyroidism (r = + 0.50, p < 0.008), but neither with serum T4 nor TSH. In a multiple regression analysis TRAb was the only significant independent variable in relation to lumbar spine BMD (F = 3. 90, p < 0.01).

**Conclusions:**

In euthyroid women with a history of Graves’ hyperthyroidism, BMD was only affected in the postmenopausal group. The negative correlation of Z-score of lumbar spine BMD with TRAb suggests that this antibody may affect the bone metabolism.

## Introduction

Thyroid hormones exert their effect on osteoblasts via nuclear receptors stimulating osteoclastic bone resorption [1-3]. Hyperthyroidism is thus one of the major causes of secondary osteoporosis.

Reduction in bone mineral density (BMD) following hyperthyroidism in female subjects has been described in many reports [4-10]. A bone histomorphometric study in patients with hyperthyroidism has shown that the increase in osteoclastic resorption was more prominent in cortical than in cancellous bone [9,11] and that normalization of thyroid function was associated with an increase in lumbar spine BMD, which was preceded by a significant attenuation of bone turnover [12]. However, discrepancy exists in the results of studies to determine whether antithyroid treatment can completely normalize bone metabolism [13,14]. In those studies, the time of follow up varied considerably, the populations were heterogeneous with reference to etiology of hyperthyroidism, osteoporosis risk factors and menopausal status.

Furthermore, it has recently been demonstrated that TSH affects bone metabolism through the TSH receptor found on osteoblast and osteoclast precursors in mice [15]. On the other hand, both higher serum TSH receptor antibodies (TRAb) and thyroid stimulating antibodies had a significant correlation with a reduction in BMD at the distal radius in male patients with untreated Graves’disease. In addition, higher TSAb significantly correlated with higher urinary N-terminal telopeptide of type I collagen [16].

Previous studies have suggested that the past history of Graves’ disease itself, and not the current state of thyroid function, was responsible for bone loss in women receiving long-term levothyroxine therapy [17]. These results suggested some deleterious effects of TRAb and TSAb on bone metabolism, probably via TSH receptors on osteoblasts or osteoclasts.

The aim of our study was to determine BMD in pre and postmenopausal euthyroid female patients with previous overt hyperthyroidism due to Graves’ disease as well as the factors that could affect BMD in each group, including TRAb.

## Materials and methods

### Subjects

One hundred and twenty-two patients with personal history of Graves’ disease and euthyroidism attended consecutively in our endocrine Division between 2006 and 2008 were evaluated. Fifty seven patients who fulfilled the inclusion criteria were consecutively enrolled in this study after informed consent. The study was carried out in compliance with the Helsinki Declaration. Sixty five patients were excluded by previous bone fracture (n = 3); non-thyroidal illness (n = 16); intake of drugs that could influence bone metabolism (n = 18); incomplete follow up (n = 21); early menopause (n = 1) and refused to participate (n = 6).

Inclusion criteria were: personal history of Graves’ disease and persistent euthyroidism for at least 6 months before entering the study. Exclusion criteria were: personal history of fracture prior to the beginning to the disease, non-thyroidal illness (liver disease, renal dysfunction, malignancy, diabetes mellitus, hyperparathyroidism, hypercortisolism, or hypogonadism) or intake of drugs (active vitamin D3, bisphosphonates, calcitonin, testosterones, steroids, diuretics, heparin, or anticonvulsants) that could influence bone metabolism and early menopause. All subjects underwent plain x-ray (anteroposterior and lateral views) of the lumbar spine, and those found to have scoliosis, compression fractures, or ectopic calcifications that could interfere with the bone mineral results were also excluded.

The diagnosis of Graves’ disease had been established by the presence of symptoms and signs of hyperthyroidism, diffuse goitre, ophthalmopathy and/or positive TRAb, high serum concentrations of thyroxine (T4) and triiodothyronine (T3) and suppressed TSH. Ultimate treatment was achieved with antithyroid drugs in five patients and radioiodine in fifty-two patients. All patients had at least two T4 and TSH values within the normal range for at least 6 months prior to this study.

The patients were divided into two groups according to their menopausal status at the moment of the study (Premenopausal n = 30 and Postmenopausal n = 27) and were compared with 52 euthyroid controls mathed according to age, gender, and anthropometrical status (Premenopausal controls n = 36 and Postmenopausal controls n = 16). Menopause was defined as one year of amenorrhea and high levels of FSH.

All subjects completed a questionnaire administered by the physician or nurse and underwent laboratory blood tests. The questionnaire determined risk factors of osteoporosis, calcium intake and score of activity (0 = immobile, 1 = normal daily activity, 2 = programmed physical activity 2 times per week, 3 = programmed physical activity 3 times per week and 4 = programmed physical activity > three times per week.

Total time of hyperthyroidism was calculated as the sum of all the periods in which the patient had high levels of thyroid hormones and suppressed TSH (including relapses). Time of euthyroidism was considered since the moment the patient permanently normalized T4 and TSH levels postreatment until inclusion in the study. The total time of evolution of the disease includes the sum of both periods: hyperthyroidism and euthyroidism.

### Biochemical measurements

Blood samples were drawn after an overnight fast in all the patients and matched controls of this study. The thyroid function variables: T4 and TSH were measured using commercially available kits (Solid phase competitive chemiluminescent enzyme inmumoasay-Immulite 2000, interassay coefficient of variation (CV): 10% and 5% respectively). TRAb was measured by radioreceptor assay [18] using a commercial kit (Radioreceptor assay; this method is based on the ability of TRAb to inhibit the binding of 125 I b-TSH to detergent-solubilized TSH-receptors from porcine thyroids cells. Results are expressed as the percentage of inhibition of 125I b-TSH binding, interassay CV: 10%). Control values were (mean +/− SD) 2.4+/−6.1% and results higher than 14.6% were considered positive [18-20]. Briefly, total calcium, phosphorus and creatinine were measured in serum using standard laboratory methods. Serum intact parathyroid hormone was measured by an Immulite 1000 with intra-and interassay CVs of 5.5% and 7.9%, respectively. Serum total 25-hydroxyvitamin D (25OHD) was measured by a DiaSorin RIA with inter- and intra-assay CVs 12,8% and 8,4%, respectively.

### BMD measurements

BMD measurements were performed at the Lumbar spine (L2-L4), hip and whole body by dual energy X-ray absorptiometry (DPX-L; Lunar, Madison, WI). Values of BMD were expressed as the mean in g/cm2 and Z-scores on the basis of normal reference values of an age and gender-matched group provided by the DXA system manufacturer. The same operator measured all the subjects. The phantom precision expressed as the CV (%) was 0.82.

### Statistical analysis

Results are expressed as percentage, mean ± SD when data passed a normality test of Kolmogorov-Smirnov or median and range when they did not. Differences between groups were analyzed using the Student T-test (when the distribution was normal) or Mann–Whitney’s U test for assessment of non-parametric median values. Correlation coefficients and multiple regression analyses were calculated. A p-value <0.05 was considered statistically significant. Statistical analysis was performed with the GraphPad Instat software program and SPSS 17.0. Advanced Calculus. Statistical Software. SPSS Inc, Chicago. 2008.

A post hoc study was applied in order to evaluate the power calculation with a 0.5 difference between the Z- score medians.

## Results

**Figure 1:**
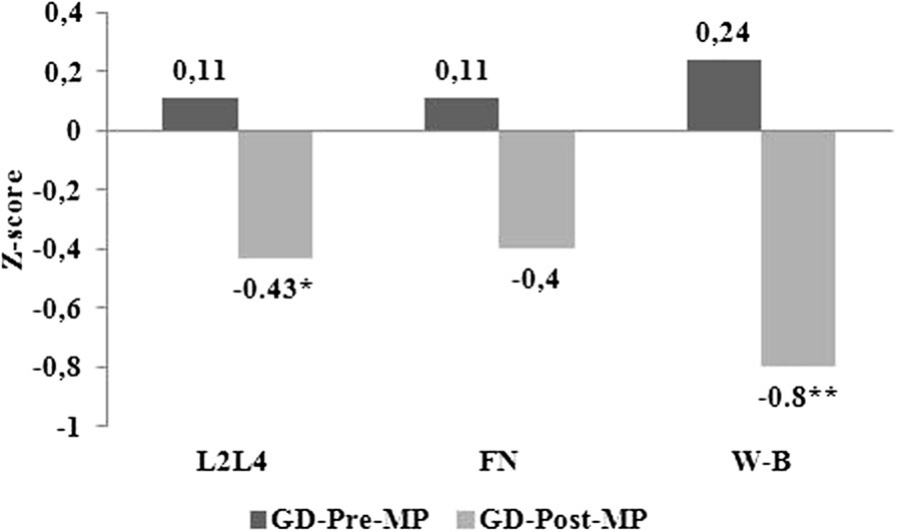
**Bone mineral density expressed as Z-score in W-B, FN and L2-L4 in premenopausal (n = 30) vs postmenopausal patients (n = 27) with a history of hyperthyroidism due to Graves’ disease.** *: p < 0,005, **: p < 0,0002. *W-B: whole body; FN: femoral neck; L2-L4: lumbar spine; Pre-MP: premenopausal; Post-MP: postmenopausal.*

The euthyroid patients with a history of hyperthyroidism due to Graves’ disease did not differ in relation to their respective controls in demographic and anthropometric characteristics, osteoporosis risk factors nor in biochemical data (Table 1). The euthyroid patients with a history of hyperthyroidism due to Graves’ disease evaluated in premenopause presented BMD similar to their respective controls in all the regions studied and postmenopausal women only presented a lower whole body BMD than controls (Table 1). Furthermore, both whole body and lumbar spine BMD Z-scores were significantly lower in postmenopausal compared to premenopausal women (Figure 1).

**Table 1:** Data of pre and post-menopausal patients with history of hyperthyroidism by Graves’ disease

	**GD-Pre-MP (n = 30)**	**C-Pre-MP (n = 36)**	**p**	**GD-Pos-MP (n = 27)**	**C-Pos-MP (n = 16)**	**p**
**Age (years)**	38,8 ± 9,7	36,3 ± 10,6	ns	56,6 ± 5,5	54,8 ± 7,4	ns
**Age of menarche (years)**	12,7 ± 1,1	12,6 + 1,2	ns	12,9 + 0,6	12,8 + 0,9	ns
**Age of menopausal (years)**	-	-	ns	47,6 ± 4,6	47,5 ± 4,7	ns
**BMI**	25,2 ± 3,8	23,2 ± 4,2	ns	26,1 ± 4,5	28,6 ± 4,7	ns
**Maternal hip fracture (%)**	0	0	ns	0	0	ns
**Fracture (%)**	0	0	ns	1 (4)	0	ns
**Tobacco (%)**	3 (10)	4 (11)	ns	2 (7,4)	4 (25)	ns
**Alcohol (%)**	0	0	ns	0	0	ns
**Physical activity**	1,3 ± 0,6	1,1 ± 0,5	ns	1,3 + 0,7	1,2 ± 0,5	ns
**Calcium intake (gr/day)**	240 (23–1113)	416 (117–1277)	ns	795 (194–1562)	280 (51–886)	<0.003
**Serum calcium (mg/dL)**	9.4 ± 0.5	9.4 ± 0.4	ns	9.4 ± 0.4	9.4 ± 0.4	ns
**Serum phosphorous (mg/dL)**	3.4 ± 0.6	3.7 ± 0.6	ns	4.0 ± 0.5	4.0 ± 0.5	ns
**Serum creatinine (mg/dL)**	0.7 ± 0.1	0.8 ± 0.1	ns	0.7 + 0.1	0.8 + 0.1	ns
**PTH (ug/mL)**	59.8 ± 30.7	45.9 ± 15.8	ns	48.3 ± 17.9	62.8 ± 24.2	ns
**25OHD (ng/mL)**	26,7 ± 11,5	50,3 ± 20,5	ns	29,7 ± 17,2	37,1 ± 12,4	ns
**BMD L2-L4 (gr/cm**^**2**^**)**	1,19 ± 0,15	1,17 ± 0,12	ns	1,001 ± 0,17	1,06 ± 0,13	ns
**BMD L2-L4 (z-score)**	0,11 ± 1,19	0,04 ± −1,0	ns	−0,43 ± 1,17	−0,51 ± 0,83	ns
**BMD FN (gr/cm**^**2**^**)**	0,96 ± 0,10	0,95 ± 0,12	ns	0,852 ± 0,14	0,84 ± 0,13	ns
**BMD FN (z-score)**	0,11 ± 0,8	0,00 ± 0,89	ns	−0,04 ± 0,87	−0,53 ± 0,81	ns
**BMD W-B (gr/cm**^**2**^**)**	1,13 ± 0,08	1,15 ± 0,06	ns	0,99 ± 0,14	1,12 ± 0,10	<0,003
**BMD W-B (z-score)**	0,24 ± 0,91	0,61 ± 0,74	ns	−0,79 + 1,03	0,40 ± 0,90	<0,0007

Data are presented as means ± SD, median or percentages. The power calculation with a 0.5 difference between the Z- score medians for Lumbar spine (L2-L4), femoral neck (FN) and whole body (W-B) were between 0.32 and 0.58. *GD-Pre-MP* Premenopausal Graves Disease, *GD-Post-MP* Postmenopausal Graves Disease, *C-Pre-MP* Premenopausal controls, *C-Pos-MP* Post-menopausal controls.

**Figure 2:**
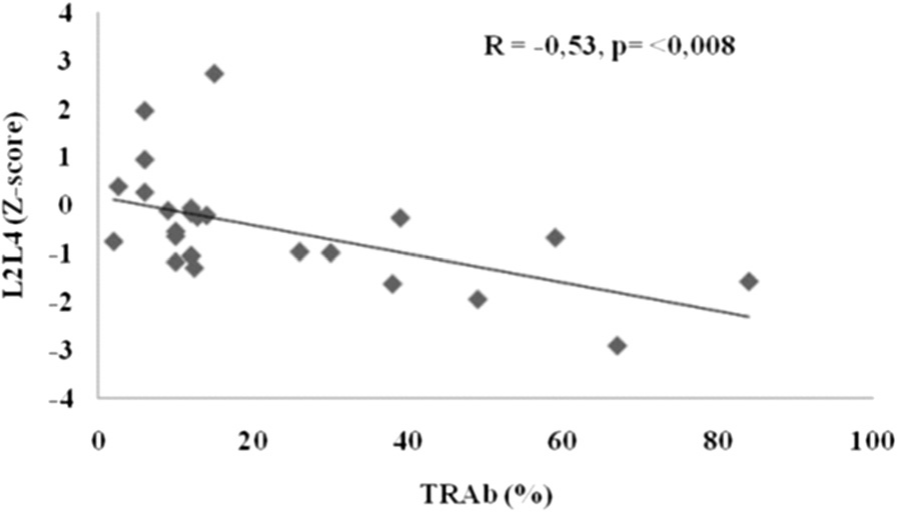
**Negative correlation between bone mineral density Z-score of L2-L4 and TRAb in postmenopausal patients with Graves’ disease (n = 27).** (R = −0, 3, p < 0,008).

**Figure 3:**
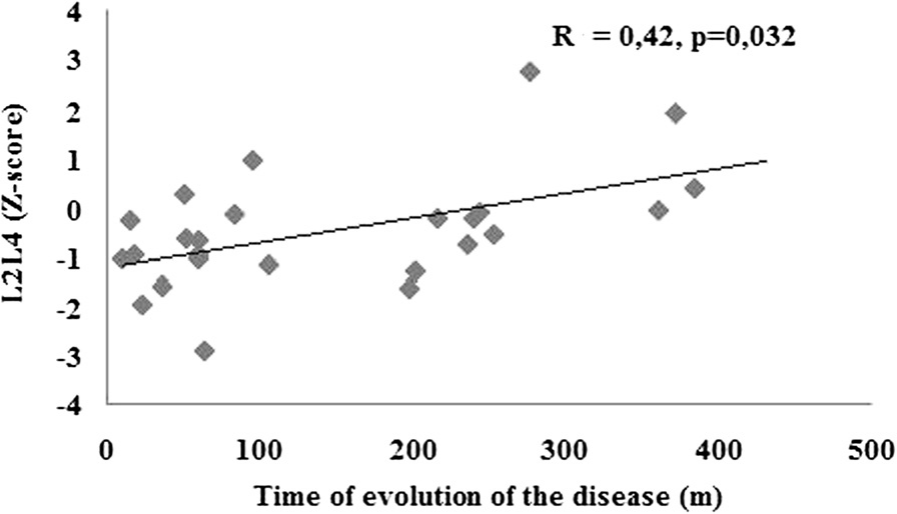
**Positive correlation between L2-L4- Z score and time of evolution of the disease (months) in postmenopausal patients with Graves’ disease (n = 27).** (R = 0, 42, p < 0,032).

**Figure 4:**
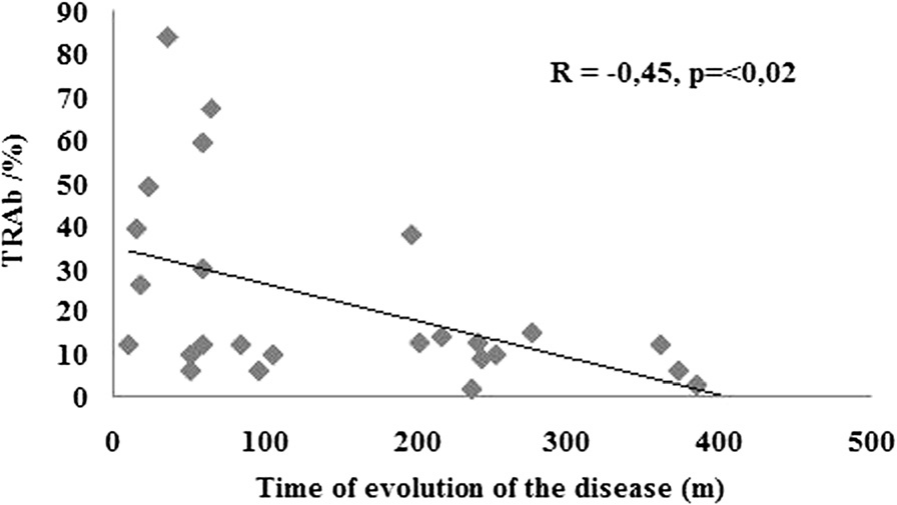
**Negative correlation between TRAb (%) and time of evolution of the disease (months) in pos-menopausal patients with Graves’ disease (n = 27).** (R = −0, 45, p < 0,02).

In order to investigate which variable could account for the smaller BMD expressed as Z-score in posmenopausal patients in relation to the premenopausal ones, both groups with previous hyperthyroidism due to Graves’ disease were compared. No significant differences were found in osteoporosis risk factors nor in biochemical data of mineral metabolism. Calcium intake was higher in the postmenopausal than in the premenopausal group (p < 0.003). The posmenopausal euthyroid Graves’ disease patients presented the beginning of their disease at an older age, had a longer time of evolution of the disease, a longer time of euthyroidism and a higher percentage of patients with L-T4 treatment than the premenopausal group. No differences were found in the levels of T4, TSH, TRAb or the percentage of patients with persistently positive TRAb at the time of the study (Table 2). In the premenopausal group, no correlation was found between the Z-score of L2-L4 with time of evolution of the disease, time of euthyroidism, nor with TRAb at the moment of study. However, in the posmenopausal group, the Z-score of lumbar spine correlated negatively with TRAb (r = −0,53, p <0.008) and positively with time of evolution of the disease (r = +0.42, p < 0.032) and with time of euthyroidism (r = 0.50, p < 0.008) (Figures 2 and 3). On the other hand, TRAb correlated negatively with the time of evolution of the disease (r = −0.45, p <0.02) (Figure 4). There was no correlation between BMD and T4 or TSH (r = +0.14 and −0.19 respectively, p:.ns). In the multiple regression analysis using the Z-Score of BMD in L2-L4 as the dependent variable and TRAb, T4, TSH and time of evolution of the disease as independent variables, the TRAb was the only significant variable in relation to L2L4 BMD, accounting for 45.2% of the variation in L2L4 BMD (F = 3,90, p <0.01).

**Table 2:** Graves’ disease characteristics in euthyroid premenopausal vs postmenopausal patients

	**G-Pre-MP (n = 30)**	**G-Pos-MP (n = 27)**	**p**
**Age of beginning of the disease (years)**	30.0 ± 7.7	40.9 ± 11.0	0,0007
**Time of hyperthyroidism (months)**	38,5 ± 44,9	53,7 ± 88,0	ns
**Time of euthyroidism (months)**	70,6 ± 68,2	146,8 ± 126,9	0,007
**Time of evolution of disease (months)**	98,8 ± 80,5	198,9 ± 130,0	0,002
**Euthyroid patients with T4 (%)**	60	93	0,003
**T4 (ug/dL)**	9,2 ± 2,2	9,8 ± 1,8	ns
**TSH (uUI/mL)**	1,4 ± 1,4	1,6 ± 1.3	ns
**TRAb (%)**	22,4 ± 19,0	21,5 ± 21,7	ns
**PositiveTRAb patients (%)**	45	34	ns

Data are presented as means ± SD, median or percentages. *GD-Pre-MP* Premenopausal Graves Disease, *GD-Post-MP* Postmenopausal Graves Disease.

## Discussion

Hyperthyroidism has been shown to accelerate bone turnover [11] and shorten the normal bone remodelling cycle [21]. Thus, active thyrotoxicosis resulted in a 12–15% reduction of BMD, predominantly in cortical bone [22]. Several small studies have been devised to assess the change in bone mass after treatment of hyperthyroidism. Although all of these studies have demonstrated improvement in bone density after restoration of the euthyroid state, the amount of improvement and the time frame evaluated varied considerably, the populations studied on the different surveys were heterogeneous with reference to etiology of hyperthyroidism, osteoporosis risk factors and menopausal status [23-27]. The long-term effects of treated thyrotoxicosis remain uncertain, but most studies suggest a persistent two to threefold increased relative risk for hip fracture, mainly in postmenopausal women [28-32].

In the present study only women with previous Graves’ disease hyperthyroidism were investigated, and they were analyzed in two groups according to the menopausal status at the time of evaluation. It was demonstrated that the population of premenopausal patients with a previous history of hyperthyroidism due to Graves’ disease and long time of euthyroidism, showed no differences in BMD in relation to their matched controls; while postmenopausal patients at the time of evaluation presented a lower whole body BMD in relation to their matched controls. Whole body BMD represents predominantly cortical bone, and it is known that thyroid hormone excess causes mainly cortical bone loss [22]. This was in keeping with results obtained in hyperthyroid patients of non-autoimmune origin such as multinodular goitre [33]. Since only three patients were excluded for prior fractures, it is improvable that exclusion of patients with more severe bone disease may have resulted in a bias of this study.

When comparing the euthyroid postmenopausal to euthyroid premenopausal Graves’ disease patients, not only did they have a lower whole body BMD Z-score but also a lower lumbar spine BMD Z-score.. The BMD difference between pre- and postmenopausal patients at the time of evaluation with a previous history of hyperthyroidism due to Graves’ disease is remarkable. This difference cannot be due to posmenopausal status per se, since it was avoided using Z score (on the basis of normal reference values of an age- and gender-matched group provided by the DXA system manufacturer), or to the duration of hyperthyroidism which was similar in both groups, or to the time of euthyroidism which was even longer in the posmenopausal group. T4, TSH and TRAb values were similar at the time of evaluation in both groups. Despite the fact that the inclusion criteria of persistent euthyroidism for 6 months or more before entering the study may have introduced a bias due to the short time interval for re-establishing normal BMD-levels, medium time of persistent euthyroidism was 70.6 and 146.8 months for pre and posmenopausal respectively.

The main difference between these groups was the ten-year later onset of hyperthyroidism in the postmenopausal group compared to the premenopausal group at the moment of the evaluation, the former starting during their perimenopause. This would reaffirm that the beginning of hyperthyroidism during this period of great vulnerability of bone mass [34] has a more deleterious effect on bone or does not allow a complete recovery of BMD.

This study shows that postmenopausal patients at the time of evaluation with a previous history of perimenopausal hyperthyroidism due Graves’ disease, despite a long time of euthyroidism, had a lower bone mass than matched controls. In contrast, those patients studied in premenopause with a previous history of hyperthyroidism due Graves’ disease did not show any difference from matched controls.

In the correlation analyses, only the postmenopausal population showed an inverse correlation between L2-L4 Z-score and the level of TRAb as well as a positive correlation with the time of evolution of the disease and time of euthyroidism. On the other hand, as expected, TRAb and time of evolution of the disease showed a negative correlation. So with longer time of evolution of the disease, lower TRAb levels and better BMD were seen. TRAb was the only significant variable in relation to lumbar spine BMD in the multiple regression analysis. This result suggested a deleterious effect of TRAb on bone metabolism. To our knowledge, only a few studies conducted to date have suggested an effect of TRAb on BMD in patients with Graves’ disease [16,35]. To our knowledge, no similar analyses were performed in women with a history of toxic multinodular goitre, i.e. subjects negative for TRAb.

A recent study on TSH receptor in null mice found evidence for direct effects of TSH on osteoblastic bone formation and osteoclastic bone resorption, mediated by the receptor on osteoblast and osteoclast precursors. These animals were found to be osteoporotic despite thyroid extract replacement therapy, linking directly the bone phenotype to the lack of action of TSH on bone [15]. Consequently, the authors suggested that the skeletal loss occurring in hyperthyroidism was due to the low TSH rather than thyroid hormone excess. In this way, the receptor antibodies could play a direct role on BMD by a similar mechanism to TSH, but the present results seem to be opposite to those reported about effect of TSH on bone turnover. This difference could be explained by different sites of action for TSH and antibodies on the TSH receptor [36]; switches in the pool of antibodies with predominance of blocking antibodies that would not allow TSH action, which cannot be discriminated with the TRAb assay performed in this study [37] or regulation of the TSH receptor by stimulating TSH autoantibodies [38]. In relation to TRAb on DMO, the study by Majima et al. [16] is in line with our results. They found that both higher serum TSH receptor antibodies (TRAb) and thyroid stimulating antibodies (TSAb) had a significant correlation with a reduction in BMD at the distal radius in male patients with untreated Graves’ disease. In addition, higher TSAb significantly correlated with higher urinary N-terminal telopeptide of type I collagen. On the other hand, Belsing et al. [39] showed that the best predictors for BMD were a negative association with free T4 and a positive one with TRAb. However, they included only premenopausal women and the follow-up was shorter than in this study.

More recently, Morimura et al. [40] showed that TSH positively regulated intracellular T3 production by controlling type 2 iodothyronine deiodinase in human osteoblasts. It is, therefore, tempting to hypothesize that TSH receptor antibodies might overproduce intracellular T3 to decrease bone mass by accelerating bone turnover.

Previous studies have suggested that the past history of Graves’ disease itself, not the current state of thyroid function, is responsible for bone loss in women receiving long-term levothyroxine therapy [17] indicating some autoimmune effects of TRAb on bone metabolism. It is known that normalization of the autoimmune abnormality comes much later than euthyroidism and the disappearance of TRAb in serum came gradually over a considerable period of time [41]. A decreased fracture risk in patients with hyperthyroidism treated with surgery and an increased fracture risk in patients treated with radioiodine [31,42] could also support this hypothesis of TRAb being involved in bone metabolism, considering that surgical treatment of Graves’ disease was associated with less pronounced or shorter elevation of TRAb, whereas radioiodine treatment was associated with a higher elevation [41].

It is not clear why the estrogen status influences thyroid hormones and TSH effects on bone mass. Both T3 and estradiol are essential for normal bone turnover in vivo, as demonstrated by the skeletal phenotypes of aromatase-deficient mice, human aromatase deficiency and postmenopausal women [43]. One recent study showed that acute TSH administration in postmenopausal women resulted in an increase of serum N-terminal propeptide of type-I procollagen, an index of osteoblastic activity, associated with an increase of serum RANKL. Lack of this response in premenopausal women suggested an influence of estrogen status on bone reactivity to TSH [44].

In our cross-sectional study, postmenopausal patients with history of Graves’ disease hyperthyroidism, with normal TSH and T4 and longstanding euthyroidism, TRAb exhibited a significant negative correlation with the Z-score of lumbar spine, suggesting that TRAb might affect bone metabolism in these patients.

The present study has several limitations, such as the small number of patients, the lack of BMD at the distal radius measurement and the fact that there were no data about bone status prior to the beginning of the disease, but patients with other causes of BMD loss were excluded. The radioreceptor assay used to assess TRAb concentrations in this study, based on inhibition of binding of ^125^I, could reflect stimulating as well as blocking activities. Assays that detect cAMP production could be useful to discriminate the antibodies activity [45,46].

The strengths include that it is the first study in Graves’ disease patients with longstanding euthyroidism, which has analyzed homogeneous subpopulations in menopausal status and osteoporosis risk factors. These results show, in relation to respective matched controls, a normal BMD in premenopausal patients with previous hyperthyroidism, but a diminished BMD in postmenopausal patients with a previous history of perimenopausal hyperthyroidism due to Graves’ disease, despite a long time of euthyroidism. They also show, in the last group of patients, an association between TRAb and BMD regardless of hyperthyroidism. We consider that these results suggest that the past history of Graves’ disease itself, and not the current state of thyroid function, could be responsible for bone loss in postmenopausal women.

This study does not show a direct causal relationship nor does it elucidate the mechanisms by which serum TRAb could affect bone measurements. Further studies will be needed to understand the differential actions of thyroid hormones, TSH and TRAb on osteoblasts and osteoclasts and their relationship with estrogens.

## Competing interests

The authors declare that they have no competing interests.

## Authors’ contributions

MAE, MLD, MCSC and ATG contributed to conception and design, acquisition of data, analysis and interpretation of data; has been involved in drafting the manuscript and revising it critically for important intellectual content; and has given final approval of the version to be published. AMF MM and FV contributed to acquisition of data, analysis and interpretation of data; has been involved in drafting the manuscript and revising it critically for important intellectual content; and has given final approval of the version to be published. UF-R has contributed analysis and interpretation of data; has been involved in drafting the manuscript and revising it critically for important intellectual content; and has given final approval of the version to be published. All authors read and approved the final manuscript.
